# Supplementary material for: Content-rich biological network constructed by mining PubMed abstracts
Source: BMC Bioinformatics. 2004 Oct 8;5:147. doi: 10.1186/1471-2105-5-147 (PMC528731; doi:10.1186/1471-2105-5-147)
Supplement: Additional File 2 — The original results of the above study (non-essential files are deleted to keep the file size under the limit set by BMC bioinformatics). [file 1471-2105-5-147-S2.bz2 › chilibotAdditionalFile2/dip50/351ID8663118E4684/html/IL13_IL13RA2.html]

 


 **IL13** and **IL13RA2** 
  
Found 82 abstracts in PubMed, retrieved 50.  
 

 What does Google say? 
 PDF only 
| .edu only 

---

**Interactive relationship** (e.g. stimulation, inhibition, etc)

**Neutral relationship**- We also observed cooperativity phenomena in IL 13  [ **IL13** ]  and IL 13R  [ **IL13RA2** ]  interaction in control RCC MLneo cells, but not in cells transfected with gammac chain MLgammac.  Ref: 10388747 Biophys J, 1999
- IL 13  [ **IL13** ]  showed a modest antitumor activity to IL 13R  [ **IL13RA2** ]  alpha 2 chain overexpressing tumors in vitro and in vivo.  Ref: 11748276 J Exp Med, 2001
- IL 4 operates through the IL 4 receptor IL 4R, a heterodimer of IL 4Ralpha and either gammac or IL 13Ralpha1 and IL 13  [ **IL13** ]  operates through IL 13R  [ **IL13RA2** ]  a heterodimer of IL 4Ralpha and IL 13Ralpha1 to promote IgE synthesis and IgE based mucosal inflammation which typify atopy.  Ref: 10699178 Hum Mol Genet, 2000
- Interleukin 13  [ **IL13** ]  receptor IL 13R  [ **IL13RA2** ]  targeted cytotoxin,  **IL13**  PE38QQR, composed of IL 13  [ **IL13** ]  and a mutated form of Pseudomonas exotoxin PE, is found to be highly and specifically cytotoxic to human solid cancer cell lines.  Ref: 11862421 Cancer Immunol Immunother, 2002
- Our third generation experiments suggest that this approach is feasible for clinical situations as intratumor administration of plasmid carrying the IL 13R  [ **IL13RA2** ]  alpha2 chain gene sensitized these tumors to systemic or regional  **IL13**  PE therapy.  Ref: 12187325 Anticancer Drugs, 2002
- We have demonstrated that primary interleukin 13  [ **IL13** ]  IL 13  [ **IL13** ]  binding protein IL 13  [ **IL13** ]  receptor IL 13R  [ **IL13RA2** ]  alpha chain plays an important role in IL 13  [ **IL13** ]  binding and internalization in the IL 13R  [ **IL13RA2** ]  system.  Ref: 10986556 Hum Gene Ther, 2000
- Analysis of cell lines Daudi and THP 1 that differentially express gamma c and IL 13R  [ **IL13RA2** ]  alpha1 showed that IL 13  [ **IL13** ]  can activate STAT6 in IL 13R  [ **IL13RA2** ]  alpha1 positive THP 1 cells but not in gamma c positive, IL 13R  [ **IL13RA2** ]  alpha1 negative Daudi cells.  Ref: 10427971 Eur J Immunol, 1999
- In fact, IL 4R alpha with IL 13R  [ **IL13RA2** ]  alpha1 is designated both as an IL 13  [ **IL13** ]  receptor and the type II IL 4 receptor.  Ref: 10534111 J Leukoc Biol, 1999
- RT PCR showed that each culture expressed IL 4R alpha, common gamma chain, and IL 13R  [ **IL13RA2** ]  alpha 1, as well as IL 13R  [ **IL13RA2** ]  alpha 2, which negatively regulates IL 13  [ **IL13** ]  signaling.  Ref: 12077271 J Immunol, 2002
- Thus, IL 13  [ **IL13** ]  cytotoxin may be a useful agent for high IL 13R  [ **IL13RA2** ]  expressing SCCHN.  Ref: 12060640 Clin Cancer Res, 2002
- IL 13  [ **IL13** ]  exerts its actions by binding to the IL 13  [ **IL13** ]  receptor IL 13R  [ **IL13RA2** ]  on target cells, which is composed of IL 13Ralpha1 and the IL 4 receptor alpha chain IL 4Ralpha.  Ref: 11145846 Cytokine, 2001
- IL 4, IL 5, as well as IL 13  [ **IL13** ]  responses were indistinguishable in control Fc and soluble IL 13R  [ **IL13RA2** ]  alpha2 Fc fusion protein treated animals.  Ref: 9916716 J Immunol, 1999
- The IL 13  [ **IL13** ]  receptor R alpha chain is a component of both IL 4R and IL 13R  [ **IL13RA2** ]  complexes.  Ref: 10686479 Exp Clin Immunogenet, 2000
- Acquisition of sensitivity to  **IL13**  PE was observed both in vitro and in vivo when IL 13R  [ **IL13RA2** ]  alpha2 transfected human tumor cells were implanted in immunodeficient animals followed by systemic or regional  **IL13**  PE therapy.  Ref: 12187325 Anticancer Drugs, 2002
- IL 13  [ **IL13** ]  receptor alpha chain 1 IL 13R  [ **IL13RA2** ]  alpha 1 is necessary for binding to IL 13  [ **IL13** ] , and the heterodimer composed of IL 13R  [ **IL13RA2** ]  alpha 1 and IL 4R alpha chain transduces IL 13  [ **IL13** ]  and IL 4 signals.  Ref: 11058569 Int Immunol, 2000
- Specific IL 13  [ **IL13** ]  receptors are results from the association between the IL 4R alpha and the IL 13R  [ **IL13RA2** ]  alpha 2 or between two IL 13R  [ **IL13RA2** ]  alpha.  Ref: 9914942 Int Rev Immunol, 1998
- IL 13  [ **IL13** ]  cytotoxin, composed of IL 13  [ **IL13** ]  and a truncated form of Pseudomonas exotoxin, targets IL 13R  [ **IL13RA2** ]  overexpressing tumor cell lines in vitro and in vivo.  Ref: 12455052 Int J Cancer, 2003
- BACKGROUND IL 13  [ **IL13** ]  receptor IL 13R  [ **IL13RA2** ]  targeted cytotoxin,  **IL13**  PE38QQR, has been shown to have very potent anti tumor activity to IL 13R  [ **IL13RA2** ]  expressing head and neck tumor cells in vitro and in vivo.  Ref: 12435859 Mol Med, 2002
- In contrast, normal human astrocytes that did not express the IL 13R  [ **IL13RA2** ]  alpha 2 gene efficiently induced Stat6 activation in response to both IL 4 and IL 13  [ **IL13** ] .  Ref: 11861389 Cancer Res, 2002
- The primary IL 13  [ **IL13** ]  receptor complex on human monocytes is believed to be a heterodimer comprised of the IL 4R alpha chain and the IL 2R gamma chain gamma c like molecule, IL 13R  [ **IL13RA2** ]  alpha1.  Ref: 10427971 Eur J Immunol, 1999
- This was in part because of the expression of a high affinity IL 13  [ **IL13** ]  binding transmembrane protein IL 13R  [ **IL13RA2** ]  alpha 2 that inhibited IL 13  [ **IL13** ]  mediated Stat6 activation by acting as a decoy receptor.  Ref: 11861389 Cancer Res, 2002
- These findings suggest that different functional responses to IL 4 and IL 13  [ **IL13** ]  by human monocytes and macrophages may result from reduced expression of gamma c and IL 13R  [ **IL13RA2** ]  alpha1.  Ref: 10534111 J Leukoc Biol, 1999
- Interleukin IL 13  [ **IL13** ]  and IL 4 inhibit proliferation and stimulate IL 6 formation in human osteoblasts evidence for involvement of receptor subunits IL 13R  [ **IL13RA2** ] , IL 13Ralpha, and IL 4Ralpha.  Ref: 11248656 Bone, 2001
- Here we demonstrate that pancreatic cancer, renal cell carcinoma, head and neck cancer, and glioblastoma cell lines that were genetically altered to express high levels of IL 13R  [ **IL13RA2** ]  alpha chain increase their binding affinity for IL 13  [ **IL13** ] , and increase their sensitivity to  **IL13**  PE38QQR by at least 6 fold to 1000 fold compared with mock transfected control cells.  Ref: 10986556 Hum Gene Ther, 2000

**Non-interactive relationship** (e.g. studied together, co-existance, homology, etc.)

- In the absence of functional IL 13R  [ **IL13RA2** ]  alpha1 and gamma c, IL 13  [ **IL13** ]  must signal through an alternative receptor complex on MDMac.  Ref: 10427971 Eur J Immunol, 1999
- IL 13R  [ **IL13RA2** ]  alpha 2, a decoy receptor for IL 13  [ **IL13** ]  acts as an inhibitor of IL 4 dependent signal transduction in glioblastoma cells.  Ref: 11861389 Cancer Res, 2002
- To study the function of IL 13R  [ **IL13RA2** ]  on brain tumor cells, we investigated the regulation of adhesion molecules by IL 13  [ **IL13** ]  as assessed by flow cytometric analysis.  Ref: 11939409 Oncol Res, 2000
- In the present study, we show that in vivo blockade of the Th2 cytokine IL 13  [ **IL13** ] , using soluble IL 13R  [ **IL13RA2** ]  alpha2 Fc fusion protein, significantly reduced the size of pulmonary granulomas in unsensitized as well as egg sensitized mice.  Ref: 9916716 J Immunol, 1999
- Expression of IL 13R  [ **IL13RA2** ]  alpha 1 evoked STAT3 activation by IL 4 and IL 13  [ **IL13** ] , and in stimulated human B cells, on which IL 13R  [ **IL13RA2** ]  alpha 1 was highly expressed, IL 4 and IL 13  [ **IL13** ]  induced STAT3 activation.  Ref: 11058569 Int Immunol, 2000
- We recently described the expression of intermediate to high affinity receptors for IL 13  [ **IL13** ]  IL 13R  [ **IL13RA2** ]  on renal cell carcinoma RCC cells.  Ref: 9816125 Clin Cancer Res, 1996
- Therefore, additional studies should be performed to determine the expression of IL 13  [ **IL13** ]  and IL 13R  [ **IL13RA2** ]  in primary clinical samples of Hodgkin s lymphoma and both agents should be further tested in vitro and in vivo as possible therapeutic agents for HL.  Ref: 11585386 Cell Immunol, 2001
- METHODS AND RESULTS The expression of IL 13  [ **IL13** ]  and IL 13  [ **IL13** ]  receptor IL 13R  [ **IL13RA2** ]  was determined by immunostaining and reverse transcriptase polymerase chain reaction in 39 cases of HD, including 17 cases with nodular sclerosis NS type, 19 cases with mixed cellularity MC, and three cases with lymphocyte predominance LP type.  Ref: 11318902 Histopathology, 2001
- We have previously reported that interleukin 13  [ **IL13** ]  receptors IL 13R  [ **IL13RA2** ]  are constitutively overexpressed on a majority of human malignant glioma cell lines and primary cell cultures.  Ref: 12481422 Mol Cancer Ther, 2002
- Human malignant glioma cell lines express high levels of interleukin 13  [ **IL13** ]  receptor IL 13R  [ **IL13RA2** ] .  Ref: 10728667 Cancer Res, 2000
- Interleukin 13  [ **IL13** ]  receptor alpha2 IL 13R  [ **IL13RA2** ]  alpha 2 chain is highly expressed on some tumor cell lines and primary cell cultures.  Ref: 11748276 J Exp Med, 2001
- However, with reduced IL 13R  [ **IL13RA2** ]  alpha1 expression and low nuclear STAT6 activity, some IL 13  [ **IL13** ]  induced responses were unaltered in magnitude in MDMac.  Ref: 10427971 Eur J Immunol, 1999
- Inflammatory cells including neutrophils and macrophages were identified in IL 13R  [ **IL13RA2** ]  alpha 2 overexpressing regressing tumors and neutrophils were found to produce IL 13  [ **IL13** ] .  Ref: 11748276 J Exp Med, 2001
- Recent progress made in the description of IL 4 and IL 13  [ **IL13** ]  receptor complex have demonstrated the existence of two types of IL 4 receptors one constituted by the IL 4R alpha and the gamma c chain, and a second constituted by the IL 4 R alpha and the IL 13R  [ **IL13RA2** ]  alpha 1 and able to transduce both IL 4 and IL 13  [ **IL13** ]  signals.  Ref: 9914942 Int Rev Immunol, 1998
- Although interleukin 13  [ **IL13** ]  receptors IL 13R  [ **IL13RA2** ]  are overexpressed on several head and neck cancer cell lines, a majority of cell lines express only low levels of IL 13R  [ **IL13RA2** ] .  Ref: 11507072 Cancer Res, 2001
- Here, we report that Jak2 is associated with IL 4R alpha, and Tyk2 is associated with the IL 13R  [ **IL13RA2** ]  alpha 1 component of the IL 13  [ **IL13** ]  receptor complex.  Ref: 12223527 J Leukoc Biol, 2002
- This was followed by successfully treating xenograft tumor bearing nude mice with IL 13R  [ **IL13RA2** ]  directed cytotoxin  **IL13**  PE38QQR.  Ref: 12471149 J Immunol, 2002
- Interleukin 13  [ **IL13** ]  receptor IL 13R  [ **IL13RA2** ]  alpha2 chain plays a key role in ligand binding and internalization.  Ref: 12808442 Gene Ther, 2003
- Coexpression of IL 13  [ **IL13** ] , IL 13R  [ **IL13RA2** ]  alpha 1 and P STAT6 is uncommon in non Hodgkin lymphomas.  Ref: 11815733 Int Arch Allergy Immunol, 2001
- IL 13  [ **IL13** ]  and the IL 13  [ **IL13** ]  specific receptor chain IL 13R  [ **IL13RA2** ]  alpha1 are frequently expressed in cHL derived cell lines and in RS cells from biopsies of cHL tissues.  Ref: 12152987 Leuk Lymphoma, 2002
- The interleukin 13  [ **IL13** ]  receptor IL 13R  [ **IL13RA2** ]  complex is composed of 2 different chains, IL 13Ralpha1 also known as IL 13Ralpha and IL 13Ralpha2 also known as IL 13Ralpha.  Ref: 11313257 Blood, 2001
- it also acts as the signaling chain for IL 13  [ **IL13** ] , but in this case the IL 13R  [ **IL13RA2** ]  alpha 1 chain conveys the bulk of the cytokine specificity.  Ref: 12165974 Roum Arch Microbiol Immunol< MedlineTA>Roum Arch Microbiol Immunol, 2001
- H RS cells of MC and NS types frequently expressed both IL 13  [ **IL13** ]  and IL 13R  [ **IL13RA2** ] .  Ref: 11318902 Histopathology, 2001
- mRNA levels for IL 13R  [ **IL13RA2** ]  alpha1, but not IL 4R alpha, were markedly decreased in in vitro monocyte derived macrophages MDMac, and with increasing time of monocytes in culture correlated with the loss of IL 13  [ **IL13** ]  regulation of lipopolysaccharide induced TNF alpha production.  Ref: 10427971 Eur J Immunol, 1999
- These receptors are composed of IL 13  [ **IL13** ]  receptor IL 13R  [ **IL13RA2** ]  alpha1, IL 13Ralpha2, and IL 4Ralpha chains.  Ref: 11939409 Oncol Res, 2000
- Although some prostate cancer cell lines express functional IL 13R  [ **IL13RA2** ] , they are not highly sensitive to IL 13  [ **IL13** ]  cytotoxin.  Ref: 11773976 Cancer Gene Ther, 2001
- To understand the mechanisms of interaction between IL 13  [ **IL13** ]  and IL 13  [ **IL13** ]  receptors IL 13R  [ **IL13RA2** ] , and the role of the IL 2 receptor common gamma chain gammac in IL 13  [ **IL13** ]  binding and processing, we have examined IL 13  [ **IL13** ]  binding kinetics, dissociation shedding, and internalization in renal cell carcinoma RCC cell lines.  Ref: 10388747 Biophys J, 1999
- The inability of BL30 B95 8 cells to respond to IL 13  [ **IL13** ]  was attributed to the loss of expression of IL 13R  [ **IL13RA2** ]  subunits alpha1 and alpha2, a finding confirmed for a number of other BL cell lines examined.  Ref: 11591117 Cell Immunol, 2001
- This best fit model predicts that 1 two different IL 13R  [ **IL13RA2** ]  are expressed on the cell membrane, 2 a minor fraction of IL 13R  [ **IL13RA2** ]  exist as microclusters homodimers and or heterodimers without exogenous IL 1 high morphological complexity of the gammac negative control cell membrane affects the cooperativity phenomena of IL 13  [ **IL13** ]  binding, and 4 a large number of co receptor molecules is present, which helps keep the ligand on the cell surface for a long period of time after fast IL 13  [ **IL13** ]  binding and provides a negative control for ligand binding via production of the high affinity inhibitor bound to IL 13  [ **IL13** ] .  Ref: 10388747 Biophys J, 1999
- In a previous study we suggested that the large number of IL 13R  [ **IL13RA2** ]  alpha 2 monomers are silent receptors, likely representing a decoy target for IL 13  [ **IL13** ] .  Ref: 10071757 J Recept Signal Transduct Res< MedlineTA>J Recept Signal Transduct Res, 1999
- To elucidate the mechanism, we utilized four head and neck cancer cell lines SCC 25, HN12, KCCT873, and YCUM911, which express high levels of IL 13R  [ **IL13RA2** ] , and IL 13  [ **IL13** ]  toxin is highly cytotoxic to these cells.  Ref: 11862421 Cancer Immunol Immunother, 2002
- Transient expression of the IL 13R  [ **IL13RA2** ]  alpha 2 transgene in nonexpressing heterologous cells inhibited not only IL 13  [ **IL13** ]  but also IL 4 mediated signal transduction and Stat6 responsive gene expression.  Ref: 11861389 Cancer Res, 2002
- In this study, we constructed wild and mutated types of human IL 13R  [ **IL13RA2** ]  alpha 1, and analyzed IL 4 and IL 13  [ **IL13** ]  signals using an IL 13R  [ **IL13RA2** ]  alpha 1 transfected human B cell line.  Ref: 11058569 Int Immunol, 2000
- However, the number of IL 13  [ **IL13** ]  positive H RS cells was statistically higher in NS type than in MC type, but the number of IL 13R  [ **IL13RA2** ]  was similar.  Ref: 11318902 Histopathology, 2001
- Several HL derived cell lines expressed IL 13  [ **IL13** ]  message and protein and message for various chains of IL 13R  [ **IL13RA2** ] .  Ref: 11585386 Cell Immunol, 2001
- Thus, these results suggest that IL 13R112D may be a useful ligand for the study of IL 13  [ **IL13** ]  interaction with its receptors or, alternatively, in designing specific targeted agents for IL 13R  [ **IL13RA2** ]  positive malignancies.  Ref: 10799519 J Biol Chem, 2000
- We have demonstrated that when interleukin IL 13  [ **IL13** ]  receptor IL 13R  [ **IL13RA2** ]  alpha2 chain, one of the two known IL 13  [ **IL13** ]  binding proteins, is introduced into cancer cells that do not express this chain the cells acquire extreme sensitivity to a chimeric fusion cytotoxin composed of IL 13  [ **IL13** ]  and a mutated form of Pseudomonas exotoxin  **IL13**  PE.  Ref: 12187325 Anticancer Drugs, 2002
- ... the  **Il13ra2**  mRNA was not detected in these cells, indicating that IL 13Ralpha1 is the major component of the IL 13  [ **IL13** ]  receptor complex in lymphohematopoietic cells.  Ref: 11003391 Immunogenetics, 2000
- IL 13  [ **IL13** ]  and IL 13R  [ **IL13RA2** ]  alpha 1, the IL 13  [ **IL13** ]  specific receptor chain, are frequently expressed by HL derived cell lines and by RS cells from biopsy material of tissues involved by HL.  Ref: 11815733 Int Arch Allergy Immunol, 2001
